# Simon says “stay in touch”: Reachability moderates the effect of irrelevant spatial congruence

**DOI:** 10.3758/s13423-026-02879-7

**Published:** 2026-04-14

**Authors:** Michael L. Paavola, J. Toby Mordkoff, Cathleen M. Moore

**Affiliations:** Department of Psychological and Brain Sciences, University of Iowa, Iowa City, IA G60 PBSB52242 USA

**Keywords:** Perception and action, Simon Effect, Stimulus-response compatibility, Action

## Abstract

The Simon Effect refers to the finding that simple left-right manual responses tend to be faster and more accurate when the response and stimulus are on the same side (congruent) compared to when they are on opposite sides (incongruent) even though the response is determined by a non-spatial attribute (e.g., color). We compared the Simon Effect with stimuli at reachable and unreachable distances within a virtual reality environment to test its dependence on the potential to interact with the stimulus. We controlled for confounds by matching image size and the viewing angle of stimuli within the environment across groups of participants. The magnitude of the Simon Effect was larger for stimuli in reachable than unreachable locations. These findings held regardless of image-size and viewing-angle conditions. This implies that task-irrelevant spatial congruence, like task-irrelevant motor affordances, is computed in ways that reflect the potential interactions with objects in a three-dimensional world.

## Introduction

Although perception and action are often studied separately, together they serve the broader function of representing the external world in the service of guiding successful action within the context of internal goals. Patterns of performance in simple tasks can reveal aspects of this interdependence. For example when participants were asked to make responses in the form of pantomimed grasps with their left or right hands to an arbitrary stimulus attribute, such as whether a picture of an object was presented upside down or right side up, if the object included a graspable component, like the handle of a mug, responses tended to be faster and more accurate when the side of the graspable part corresponded to the side of the grasp response (e.g., Costantini et al., 2010, 2011; Joy et al., 2022; Tucker & Ellis, 1998). This motor-affordance congruence effect occurred despite the fact that the graspable part of the object and its location were irrelevant to the task which was determined by the orientation of the pictured object.

Task-irrelevant congruence effects also occur with stimuli that have no strong motor affordance that is related to the required response. For example, when people perform a task that requires a spatial response, such as a button press with the left or right hand, to a non-spatial attribute of a stimulus, such as whether it is red or green, performance is affected by the location of the stimulus even though it is not relevant to the task. Specifically, participants tend to be faster and more accurate when the stimulus is presented closer to the correct response, a pattern referred to as the *Simon Effect* after Simon and Rudell (1967). Notice that in the case of the Simon Effect, the location of the stimulus is completely irrelevant to the task and the stimulus can be as arbitrary as a colored circle on a flat computer monitor and thus afford no specific response, and yet responses are systematically influenced by its location.

Over the past 50-plus years, many details of the Simon Effect have been explored and clarified. For example, it has been shown that the apparent location of the stimulus is more important than its actual location (Simon et al., 1970), and the relative locations of stimuli across trials are more important than their absolute locations (e.g., Nicoletti & Umilta, 1994; Proctor et al., 1993). Relatedly, it has been shown that both the location of the stimulus relative to the focus of attention and its location relative to fixation can be sources of Simon Effects (Lleras et al., 2004; Nicoletti & Umilta, 1994). It has also been found that centrally presented stimuli that evoke the sensation of motion (e.g., Bosbach et al., 2004) or imply a direction (e.g., Luo & Proctor, 2018; Proctor et al., 1993) can be the source of a Simon Effect. Similarly, we know that the relative locations of the buttons are more important than the effectors that are used to make the responses (e.g., Brebner et al., 1972). And finally, the locations of the expected consequences (or “action effects”) of the responses can be as important as the locations of the physical responses made by the participants (e.g., Grosjean & Mordkoff, 2002).

The earliest explanation for the Simon Effect referred to “an innate tendency to respond towards the source of stimulation” (Simon, 1969). More modern accounts have sought to specify processing mechanisms underlying the effect (Kornblum, 1994; Proctor, 2011). A tendency to act toward a stimulus is similar to the explanation of the motor-affordance congruence effects with graspable object parts described above. Those explanations were grounded in the ecological or “neo-Gibsonian” approach to human perception and performance which stresses the interaction between people and their local environment (see, e.g., Heft, 2012; Lobo et al., 2018; Segundo-Ortin & Raja, 2024, for reviews). In the case of the Simon Effect, however, stimuli have no specific motor affordance, other than being in a particular location in space relative to the observer, which is not directly related to the required response. Therefore, the explanation of the Simon Effect in terms of potential interactions with objects in the environment asserts an extremely fundamental nature of the interdependence of perception and action.

Consistent with ecological explanations of motor-affordance congruence effects, several studies have confirmed that they depend on actors perceiving the affordances as true affordances. For example, several studies found that the congruence between the graspable part of an object and the side of the participant’s grasping response affected performance only when the object was perceived as close enough to reach (Costantini et al., 2010). Moreover, the congruence effect for objects at reachable distances was eliminated when the objects were perceived as being behind a physical barrier and therefore could not be interacted with (Costantini et al., 2010). Related, in a study using virtual reality, participants were given avatars that either did or did not possess the ability to make the specific grasp response that objects afforded. The congruence effect occurred only for those participants with avatars that possessed the ability to grasp them (Joy et al., 2022). Finally, providing a tool that extends one’s reach, whether directly to the participant or to another actor in view of the participant, can cause otherwise unreachable objects to elicit motor-affordance congruence effects (Costantini et al., 2011). These and related results (Brockmole et al., 2013) support the assertion that the perceived affordances of specific actions on objects in the world influence the computation of responses, whether the affordances are directly task relevant or not, perhaps by altering one’s perception of space (Witt, 2021) or by remapping one’s body schema (Cardinali et al., 2009). It is important to note, however, that some of these effects may not reflect automatic action priming and may be more context dependent. For example, Yu et al. (2014) showed that action priming by pictures of objects was limited to explicitly imagining interacting with said objects.

In the current study, we tested the hypothesis that the Simon Effect is similarly dependent on the perceived ability to interact with the stimulus. Specifically, we tested the prediction that the Simon Effect would be smaller with stimuli that are perceived as being unreachable compared to stimuli that are perceived as reachable. Such a finding would indicate that spatial congruence, even when stimuli possess no motor affordance that is directly related to the required response, is computed based on potential interactions with objects in a three-dimensional world. This in turn would imply an especially fundamental nature of the interdependence of perception and action.

## Logic and overview

Reachability was operationalized in the current study by distance from the participant. Specifically, a location was said to be reachable if it was within the range of one or both hands without moving the torso and unreachable if not (cf. "peripersonal space," which is not limited to the hands; Rizzolatti et al., 1997). Like any specific operationalization, there are limitations to this one. First, there are other ways through which reachability can be manipulated that might impact performance differently. Examples include introducing an apparent barrier between the actor and the stimulus (Costantini et al., 2010), providing tools that extend one’s reach (e.g., Brockmole et al., 2013; Costantini et al., 2011), and limiting the real (Moisello et al., 2008; Toussaint et al., 2020) or virtual (Joy et al., 2022) type of action an actor is capable of executing. In addition, by operationalizing reachability on the basis of distance from the observer, reachable stimuli appear closer to the participants’ hands than unreachable stimuli, which could introduce perceptual or attentional differences between conditions (Abrams et al., 2008). These limitations are considered in more depth in the *General discussion* where we argue that while they raise multiple interesting questions for follow-up work, they do not alter the main conclusions that can be drawn from the current study.

**Fig. 1:**
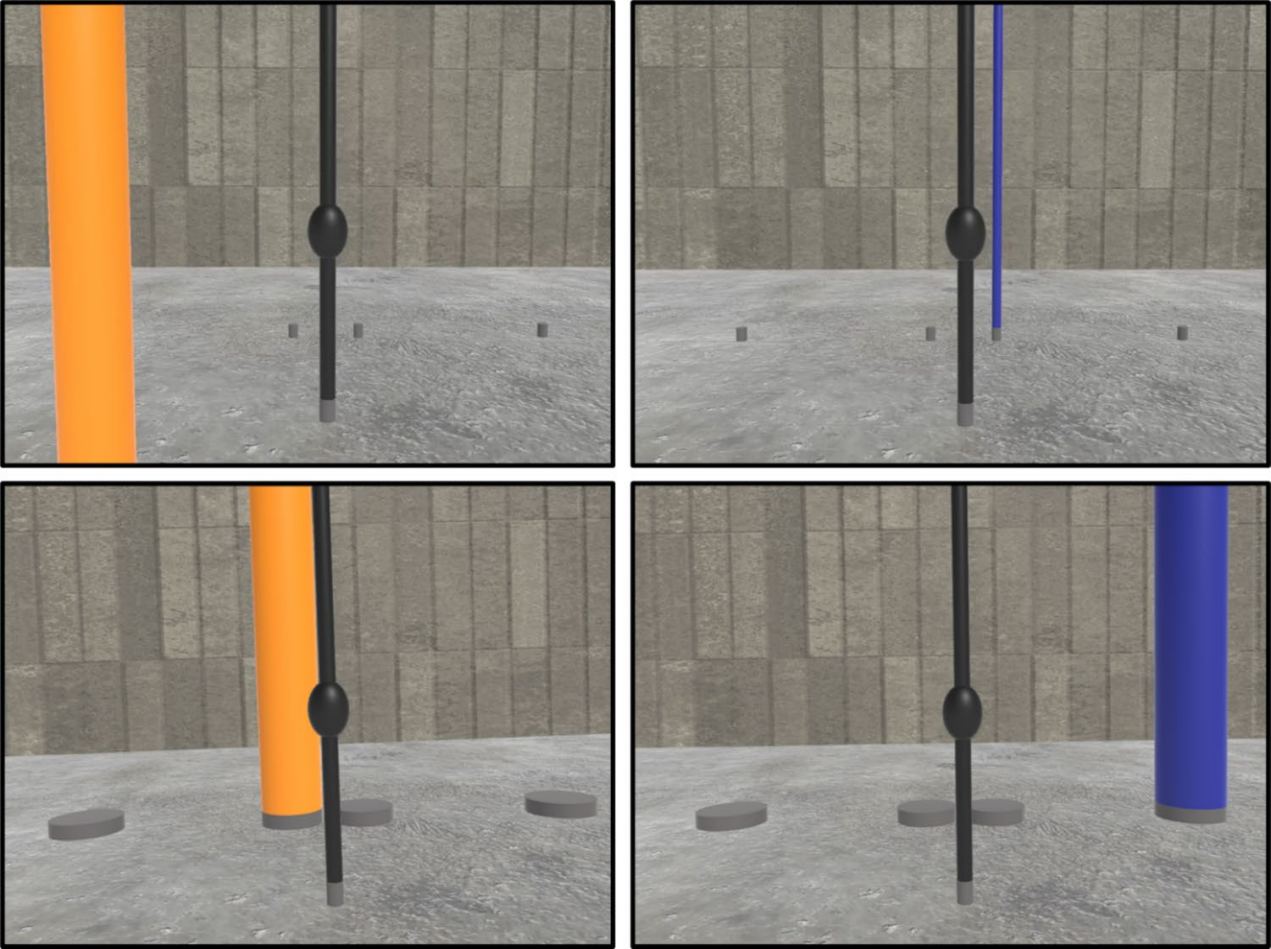
Examples of a participant’s view of the virtual environment. The upper-left panel shows a scene in which an orange pole appears on the left at a reachable distance and the upper-right panel illustrates a scene in which a blue pole appears on the right at an unreachable distance. The lower-left panel shows an example of a scene in which a pole is in an unreachable location but the image size of the stimulus matches that of a pole at a reachable location to control for decreasing retinal-image size that occurs with distance. The lower-right panel shows an example of a scene in which a pole is in an unreachable location to the right, but its angular separation matches that of a pole in a reachable location to the right to control for differences that occur with compression within the retinal image that occurs with distance

We used a virtual environment to present stimuli at reachable and unreachable locations and measured the Simon Effect. The task required a right- versus left-hand response to the non-spatial stimulus attribute of color. The locations of the stimuli were task irrelevant, but their congruence with the correct response varied by appearing to the right and left of the midline. The stimuli were colored poles that extended from the virtual ground plane at different depths from the viewer on the left or right side of the midline. Figure 1 illustrates four sample scenes. Our prediction was that reachable stimuli would produce a larger Simon Effect than unreachable stimuli.

**Fig. 2:**
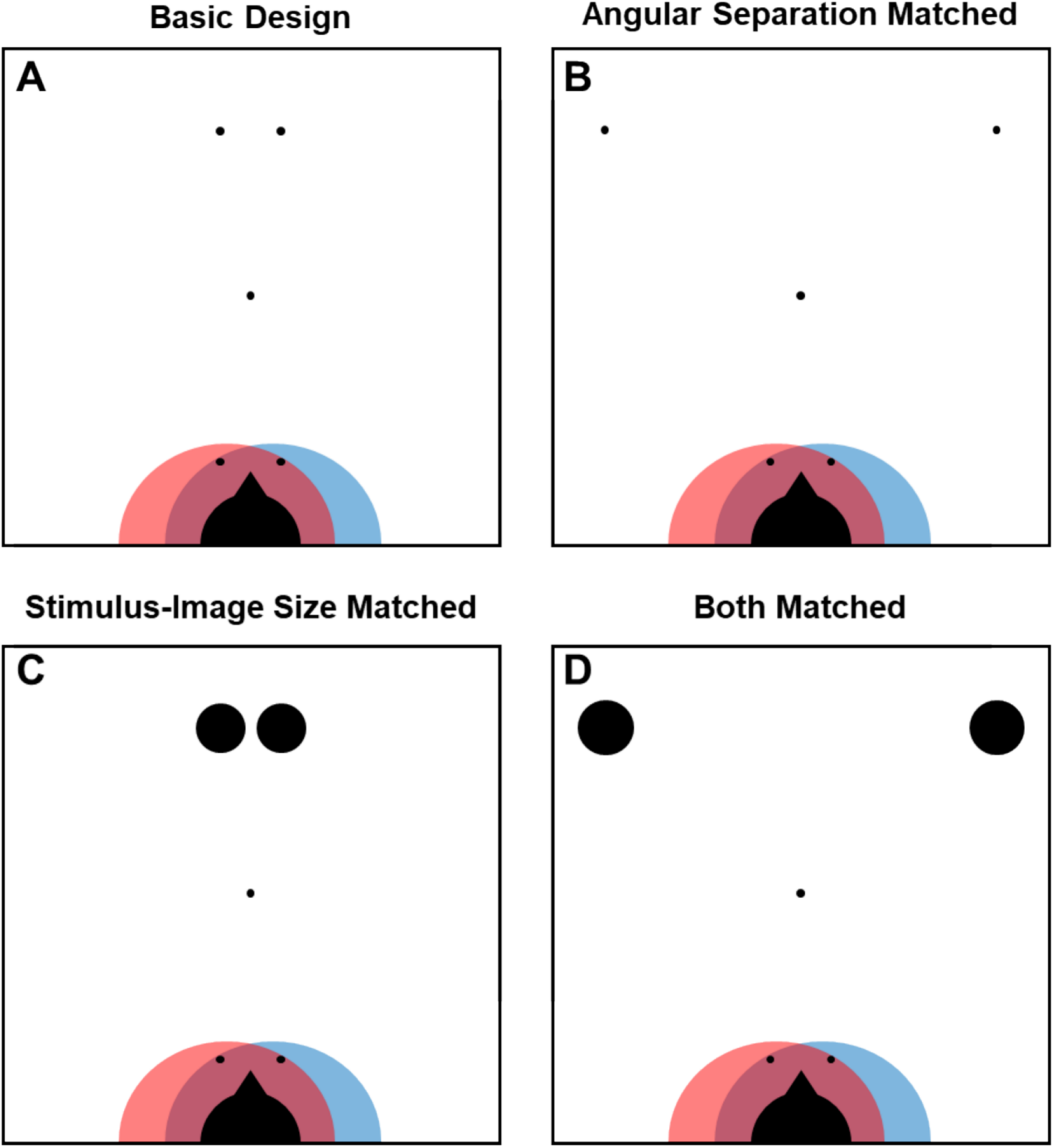
Top-down views for the four versions of the design. In each panel, the half-circle and triangle nose at the bottom shows the participant’s point of view; note that these are enlarged in order to make them more visible, whereas all other aspects of the figure are to scale. The red and blue zones indicate the range of locations within reach of participants left and right hands, respectively. The single center-most dot shows the location of a fixation pole, and the other four dots indicate reachable and unreachable stimulus locations. **Panel A** illustrates the basic design. The separation between locations and the diameter of the poles in world space were identical at reachable and unreachable locations. This resulted in different angular separations between locations and different stimulus-image sizes at reachable and unreachable locations. **Panel B** illustrates the design in which the angular separation between stimuli was the same at reachable and unreachable locations. This resulted in a much larger separation in world space between stimuli in unreachable locations than between stimuli at reachable locations. **Panel C** illustrates the design in which stimulus-image size was the same at reachable and unreachable locations. This resulted in much larger-diameter poles in unreachable locations than reachable locations. **Panel D** shows the design in which both angular separation and stimulus-image size were the same at reachable and unreachable locations

Figure 2 illustrates the overall design of the study. Panel A shows the basic design, and panels B–D show variations that controlled for different confounds in the basic design. These designs were run with different groups of participants. First consider panel A. It shows a top-down view of the locations in which stimuli could appear relative to the observer. At the bottom of the panel is the participant’s head (i.e., the filled half-circle with a nose facing toward the top of the panel). The red and blue zones indicate the range of locations within reach of the left and right hands, respectively. The five dots are the locations at which poles appeared. The single central dot is where a fixation pole was presented. The four other dots show the locations at which target stimuli (i.e., colored poles) appeared; only one colored pole appeared on a given trial. As can be seen, the two dots closer to the participant are both within reach of both of the hands, while the two farther dots cannot be reached by either hand.

Because reachability was manipulated by changing the distance of stimuli from the viewer, two potential confounds had to be addressed. The first concerns the distinction between the stimulus locations in three-dimensional (3D) world space versus the angular separation of those same locations relative to the viewer which determines their separation in the image at the eye. Specifically, in the basic design shown in Panel A of Fig. 2, the angular separation of stimuli at locations farther from the viewer is smaller than that for stimuli that are closer to the viewer. This is why linear perspective cues, like converging lines, and texture gradients are effective monocular cues to depth; they mimic the spatial compression that occurs with distance when projecting a 3D world onto a two-dimensional (2D) retina. In the basic design (Panel A), any reduction in the Simon Effect at unreachable distances could be attributed to the reduced angular separation of the stimuli rather than to their reachability. Panel B of Fig. 2 illustrates a variation of the basic design in which the angular separation of stimuli in the unreachable locations is matched to that of stimuli at the reachable locations. Notice that doing so causes the separation between the two potential stimulus locations in 3D world space to be much greater for stimuli at unreachable locations than reachable locations.

The second potential confound in the basic design concerns the distinction between the sizes of objects in the world and the sizes of the images they project to the eye. Specifically, the images of stimuli that are farther from the viewer are smaller than those for stimuli that are closer. Again, any difference in the Simon Effect for unreachable stimuli that occurs in the basic design shown in Panel A of Fig. 2 could be attributed to reachable stimuli projecting larger, and therefore potentially more intense, images than unreachable stimuli. Panel C illustrates a variation of the basic design in which the image size of stimuli at unreachable locations is matched to that of stimuli at reachable locations. Notice that doing so causes objects at unreachable locations to appear much larger than objects at reachable locations.

Finally, Panel D of Fig. 2 illustrates a design in which both angular separation and stimulus-image size of stimuli at unreachable locations were matched to those of stimuli at reachable locations.

If either of these two factors – angular separation or stimulus image size – is the source of a reduction in the Simon Effect at unreachable locations, rather than reachability being the source, then that effect should be substantially reduced or eliminated in one or more of the designs illustrated in Panels B–D. On the other hand, if the Simon Effect depends on stimuli being reachable, then it should be larger for stimuli in reachable locations than in unreachable locations in all four designs.

## Method

### Participants

A total of 96 participants (63 female, 33 male; mean age 18.6 years) completed the experiment, 24 in each of the four designs. All participants were University of Iowa undergraduates who received credit toward a research-exposure requirement in an introductory psychology course. All reported normal or corrected-to-normal visual acuity and full color vision. All procedures were approved by the University of Iowa Institutional Review Board. The number of participants was based on the effect size, *f*^*2*^ = 0.173, observed in a pilot study (*N* = 48) that roughly matched the method shown in Fig. 2, which indicated a total of 96 participants needed to achieve 80% power.

### Apparatus

Stimuli were presented using an HTC Vive Pro Eye VR headset with screen dimensions of 1,440 x 1,600 pixels per eye. The refresh rate was 90 Hz, and the field of view was 110°. Two HTC Vive controllers with SteamVR Tracking sensors were used to interact with the virtual environment. Stimulus presentation and participant responses were collected using a custom VR application written in Unity (Version 2021.3.3f1). Two base stations, at opposite corners of the room, sent signals to the headset and received data from the controllers.

### Stimuli

The VR environment consisted of a 20-m x 20-m room with walls that were 29 m high, but no ceiling. The walls were textured to appear like a set of gray concrete slabs and the floor was light gray, speckled concrete. The target stimuli consisted of colored 3D cylinders rendered in Unity, which could be shown in any of four locations in the environment. All objects used in this experiment are located at coordinates X (width), Y (height), Z (depth) referenced as [#, #, #]. A gray fixation point cylinder was positioned at [0, 0, 0] and also served as a reference point. The participant’s head position was located at position [0, 0, −2.8], which is 2.80 m in front of fixation, with each of their hands being approximately.35 m closer to the fixation. For all four groups of participants, the near locations were positioned at [-.35, 0, −2] and [.35, 0, −2], which are both 0.35 m to one side of the midline. In the equal-spatial-separation conditions (see Panels A and C of Fig. 2), the far locations were positioned at [-.35, 0, 2] and [.35, 0, 2], which are, again, 0.35 m to each side of the midline. In the equal-visual-angles conditions (Panels B and D), the far locations were located at [−2.35, 0, 2] and [2.35, 0, 2], which are 2.35 m to each side of the midline. The fixation cylinder was 10 cm in diameter and 20 m tall with a 25-cm “fixation sphere” on the pole at eye level. When expanded (as the warning signal), the fixation sphere briefly increased to 35 cm in diameter. The target cylinders varied in size depending on the condition. In the equal-actual-size conditions (Panels A and B), all cylinders were 10 cm in diameter and 20 m tall. In the equal-retinal-image condition, the far cylinders were either 60.4 cm (see Panel C of Fig. 2) or 67.3 cm in diameter (Panel D). In all conditions, there were visible placeholders on the floor at the base of each possible target location. The placeholders were slightly larger than the corresponding cylinders. Hand-check stimuli consisted of two green cylinders, which turned turquoise when touched. Each was 10 cm in diameter and 20 m tall and were presented at near positions. Participants would be excluded if unable to reach the cylinders during this phase; however, all participants were reported to have been able to touch the poles. All instructions were presented on varying-sized canvases just in front of the fixation pole.

### Tasks

The task was to report the color of the target cylinder as either orange or blue by pulling the trigger on the HTC Vive controller held in the right or left hand, respectively. The instructions emphasized that responses should be made as quickly as possible while avoiding too many errors, which was described as about three per block. Before each block of trials, participants completed a hand-check in which they reached out and touched the two reachable cylinders. Finally, after completing the main experiment, each participant completed a maximum reach task by reaching out and indicating their maximum reach via trigger pull.

### Design

This experiment is best thought of as using a two-level, within-subjects design – reachable versus unreachable – with the dependent measure being the Simon Effect, but with four different groups created by crossing the method of equalizing separation (3D space vs. visual angle) with the method of equalizing stimulus size (actual vs. retinal image) as illustrated in Fig. 2. For all four groups, there were equal numbers of trials across reachability (reachable vs. unreachable), stimulus side (left vs. right), and stimulus color (orange vs. blue). Thus, stimulus location was not predictive of stimulus color and, therefore, not predictive of the correct response. The basic design was, therefore, a standard, two-option Simon Task, conducted using VR, with the addition of reachability.

### Procedure

Each participant was tested in a single session. After providing informed consent, participants were moved to the VR room and positioned in a chair with left and right armrests approximately .70 m apart. After the experimenter initiated the VR application, they assisted the participant with putting on the VR headset. Participants were then given the two VR controllers and were shown where the trigger buttons were located and were told that no other buttons were being used for this study. They were asked to lean against the back of the chair and leave their arms positioned on the arm rests through each block of trials. They were able to change positions and rest between blocks. Participants were then given task instructions.

Before each block, participants completed a “hand check.” Two green poles were presented in the two reachable locations. Participants reached out and touched the two poles with their two hands. When they made contact, the controllers vibrated, and the poles changed to turquoise. Participants then moved their arms back to the armrests of the chair and could initiate the block by both trigger buttons.

Individual trials were also initiated by pulling both trigger buttons. A fixation pole was immediately displayed for 2,000 ms (180 frames) before the central fixation sphere expanded for ~156 ms (14 frames) before disappearing for ~356 ms (32 frames). At that point, the colored target cylinder appeared at one of the four locations indicated by the placeholders on the ground. Participants were instructed to pull the right trigger button if the target cylinder was orange and to pull the left trigger if the cylinder was blue. Once either trigger was pulled, the cylinder would disappear. If the response was incorrect, the word “incorrect” was displayed for 2,000 ms (180 frames).

Participants completed a practice block of 16 trials, followed by 15 blocks of 48 trials. The data from the first two full blocks were considered practice and were not included in analyses. This resulted in 156 observations in each of the four main conditions (i.e. Unreachable Congruent, Unreachable Incongruent, Reachable Congruent, Reachable Incongruent) that were used to calculate the Simon Effects. Participants could rest as much as they liked between blocks of trials. After finishing the final block of trials, participants completed a final task to determine their maximum reaching distance. They were instructed to lean forward in the chair with their hands outstretched as far as possible while remaining seated. Once they were stretched out as far as possible, they were instructed to pull one of the trigger buttons. After pulling a trigger button, they were instructed to sit back before being released from the experiment. The entire session lasted approximately 1 h.

### Data analysis

Responses faster than 200 ms or slower than 2,000 ms were omitted (0.19% were too fast and 0.10% were too slow). In addition, 0.41% of trials were excluded due to a minor glitch in the custom program used. Trials on which an error was made were excluded from the response-time analysis. Data collapsed over color (and, therefore, response) so that a single Simon Effect (i.e., Incongruent – Congruent) was calculated for each of the reachable and unreachable conditions in both response time (RT) and error rate (ER). Prior to ANOVA, equal variance across groups was verified for each measure using Levene’s test: all *p* ≥.059. Effect size is reported as adjusted partial eta-squared (*adj* ${\eta }_{p}^{2}$), which corrects for the positive bias inherent in standard statistic (Mordkoff, 2019).

## Results

We verified the manipulation of reachability in the virtual environment through two tests. Stimuli in the reachable locations were confirmed by the reach check at the beginning each block. If stimuli in the reachable locations were not actually reachable, the participant could not have continued. Stimuli in the unreachable locations were confirmed by measuring a subset of participants’ maximum reaches (90 subjects), which ranged from.83 m to 1.49 m, with a mean of 1.16 m. Six subjects’ maximum reaches were thrown out due to missing data or a response error in which participants indicated their maximum reach prior to their actual reach. This confirms that none of the participants could reach stimuli in the unreachable locations which were approximately 2.8 m from the participant.

**Fig. 3:**
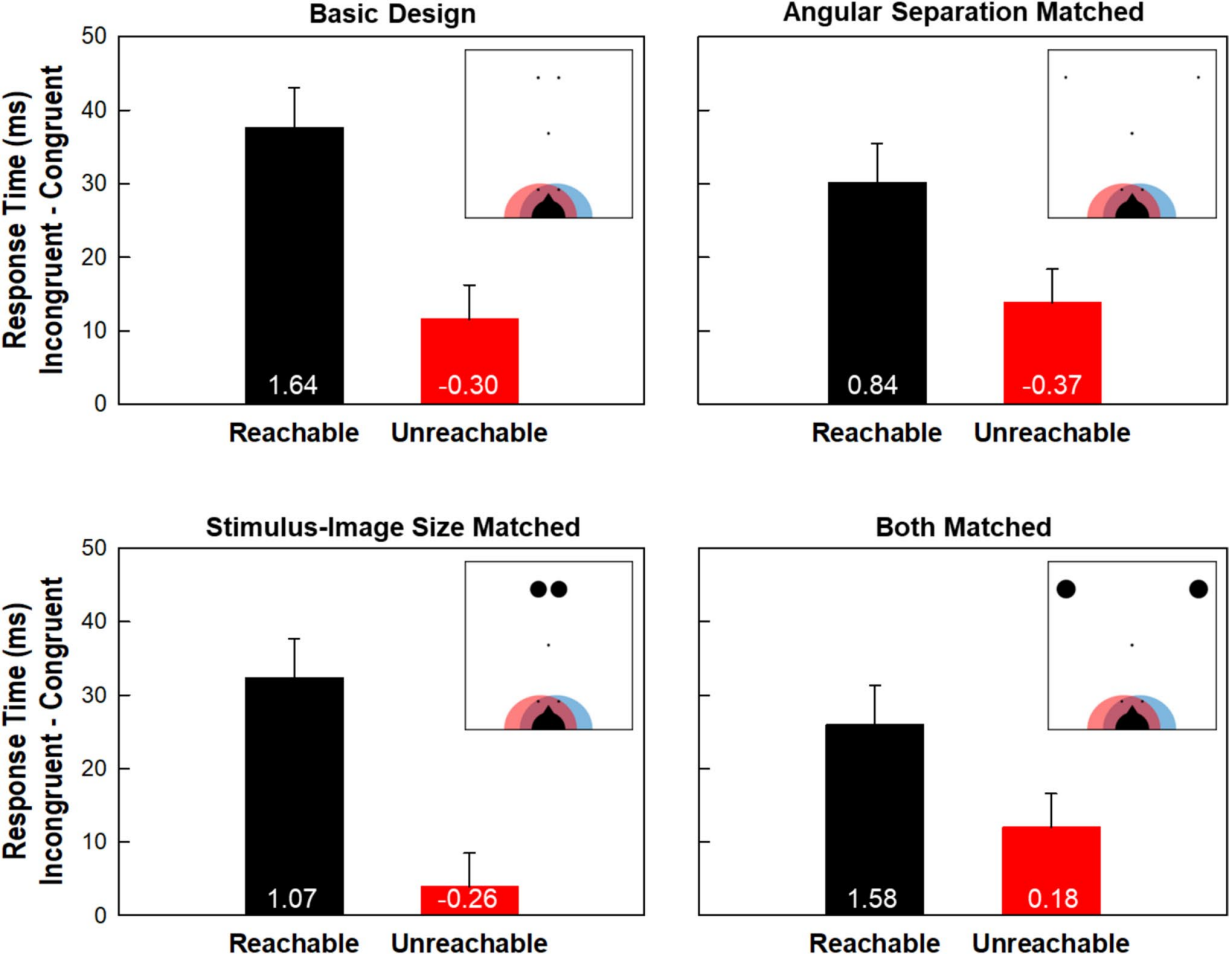
Results from all four groups. The numbers on each bar indicate the Simon Effect in error rate (ER; %). Error bars indicate the standard error of the mean (SEM) across groups. SEMs for ERs were 0.38 and 0.43 for Reachable and Unreachable conditions, respectively

Simon Effects are summarized in Fig. 3. The bars show the Simon Effects in RT and the numbers on each bar indicate the Simon Effect in ER (%). Subject data were submitted to a 2 × 2 × 2 mixed ANOVA with one within-subject factor (reachability) and two between-subject factors (separation and stimulus size). Focusing on RT, the Simon Effect was consistently larger for reachable (mean: 31.53 ± 2.69 ms) than unreachable (mean: 10.34 ± 2.29 ms) stimuli. This main effect of reachability was highly significant: *F*(1, 92) = 52.97, *p* <.001, *adj* ${\eta }_{p}^{2}$ =.359. Moreover, for all four groups, the Simon Effect was larger for reachable than for unreachable stimuli: all *t*(23) ≥ 2.93, *p* ≤.008.

With regard to the between-subject factors, again focusing on RT, neither main effect nor the interaction was significant. Specifically, there was no effect of matching separation distance across reachable and unreachable locations based on 3D distance versus angular separation: *F*(1, 92) = 0.05, *p* =.818, *adj* ${\eta }_{p}^{2}$= -.010. There was also no effect of matching the sizes of stimuli across reachable and unreachable locations based on object size versus stimulus-image size: *F*(1, 92) = 1.37, *p* =.244, *adj* ${\eta }_{p}^{2}$ =.004. And finally, the Simon Effect did not depend on the combination of these two factors: *F*(1, 92) = 0.18, *p* =.671, *adj* ${\eta }_{p}^{2}=-.009$.

For the mixed-factor effects, the effect of reachability did not depend on the equating object size versus stimulus-image size: *F*(1, 92) = 0.00, *p* =.996, *adj* ${\eta }_{p}^{2}= -.011$. It did, however, differ depending on whether separation was equated based on world space or angular separation: *F*(1, 92) = 4.32, *p* =.040, *adj* ${\eta }_{p}^{2}=.035$. Specifically, the difference between reachable versus unreachable stimuli was smaller when the angular separations were equal (mean = 13.96 ± 4.13 ms) than when the world-space separations were equal (mean = 28.45 ± 6.00 ms). Critically, however, the effect of reachability on the Simon Effect was reliable in all cases, and the three-way interaction was not significant: *F*(1, 92) = 0.17, *p* =.683, *adj* ${\eta }_{p}^{2}=-.009$.

Finally, it is worth noting that the Simon Effect for reachable stimuli did not differ as function of group: *F*(3, 92) = 0.83, *p* =.482, *adj* ${\eta }_{p}^{2}=-.005$. This is important because the locations and sizes of stimuli in reachable locations were the same for all groups (see Fig. 3, see Table 1 for full data).

**Table 1:** Response times (RTs; ms) and error rates (ERs; %) for all conditions with standard errors in parentheses. The Simon Effect is the calculated difference between Incongruent – Congruent. Bold font indicates that a given Simon Effect was significant in a one-sample *t*-test against zero

		Reachable			Unreachable		
		Congruent	Incongruent	Simon Effect	Congruent	Incongruent	Simon Effect
**Basic Design**	**RT (ms)**	497.32 (19.42)	534.98 (19.05)	**37.66** (5.37)	523.06 (20.28)	534.69 (19.41)	**11.62** (4.59)
	**ER (%)**	0.73 (0.17)	2.37 (0.36)	**1.64 **(0.38)	1.37 (0.30)	1.08 (0.19)	−0.30 (0.43)
**Angular Separation Matched**	**RT (ms)**	469.93 (19.42)	500.06 (19.05)	**30.13** (5.37)	507.08 (20.28)	520.90 (19.41)	**13.82** (4.59)
	**ER (%)**	1.80 (0.35)	2.64 (0.52)	**0.84 **(0.38)	2.23 (0.58)	1.86 (0.35)	−0.37 (0.43)
**Stimulus-Image Size Matched**	**RT (ms)**	503.7 (19.42)	536.08 (19.05)	**32.38** (5.37)	519.82 (20.28)	523.75 (19.41)	3.93 (4.59)
	**ER (%)**	1.65 (0.35)	2.72 (0.49)	**1.07 **(0.38)	2.05 (0.38)	1.79 (0.36)	−0.26 (0.43)
**Both Matched**	**RT (ms)**	523.31 (19.42)	549.23 (19.05)	**25.93** (5.37)	542.67 (20.28)	554.64 (19.41)	**11.97** (4.59)
	**ER (%)**	1.44 (0.28)	3.02 (0.56)	**1.58 **(0.38)	2.15 (0.38)	2.32 (0.43)	0.18 (0.43)

All of the analyses that were conducted on the RTs were also conducted on the ERs. No significant effect that was in a different direction from that in the RTs was revealed, indicating that none of the critical patterns of results was obscured by a speed-accuracy trade off.

## Discussion

In this study, we used virtual reality to test the hypothesis that the perceptual-motor phenomenon known as the Simon Effect depends on the reachability of the stimuli to which responses are being made. The task was to report the color of a stimulus (in this case a pole) by making a corresponding right or left manual response (in this case a trigger-pull on a controller). Poles appeared to the right and left of the participant’s midline across trials. The location of a pole was completely irrelevant to the task and yet when it was congruent with the correct effector side, responses were faster than when it was incongruent. This is a standard Simon Effect measured in a virtual environment. The critical manipulation was whether the stimulus poles were presented at a reachable or unreachable distance from the observer, confirmed by interactive tests within the virtual environment.

The results were clear. The reachability of the poles affected the magnitude of the Simon Effect in every version of the design that was tested. Specifically, when stimuli were out of reach, the influence of task-irrelevant spatial congruence on RT was reduced by approximately two-thirds. Put more generally, when participants had no possibility of interacting with a stimulus, the interaction between perception and action was greatly attenuated. Importantly, this reduction in the size of the Simon Effect held regardless of the specific method employed to control for differences in angular separation and/or image size, both of which systematically change with distance from a viewer. This reinforces the understanding of the Simon Effect as something that is driven by perceived objects in the world toward which actions can be made, rather than sensory interactions (Bosbach et al., 2004; Simon et al., 1970).

As reviewed in the *Introduction*, action affordance congruence effects have also been found to be modulated by whether or not the object that affords the specific action is reachable or not (Costantini et al., 2010; Joy et al., 2022). Moreover, the perception of the possibility of other people in the environment being able to reach stimuli or not reach them can modulate congruence effects (Costantini et al., 2011; Guangnano et al., 2010; Iani et al., 2021). The current findings contribute to this literature by demonstrating that even congruence effects where the congruence is between a task-irrelevant attribute and a reponse that is unrelated to any specific action affordance provided by the stimuli is nonetheless modulated by whether or not the stimuli are perceived as being within a range that they could, in principle, be acted upon. This, along with the previous findings, contributes to the broader idea that human information processing concerns interactions between the actor and the local environment, as opposed to being abstract (Barsalou, 2008; Brockmole et al., 2013; Costantini et al., 2010, 2011; Joy et al., 2022; Segundo-Ortin & Raja, 2024; Warren, 2006).

A limitation of the current study is that the reachable stimuli were both reachable and physically closer to the observer than the unreachable stimuli were. Prior related research dissociated effects of reachability and proximity by, for example, introducing a barrier between the participant and distant stimuli (e.g., Costantini et al., 2010), providing tools that extend participants’ reach (see Witt, 2021, for a review), or by limiting the range of actions that participants were able to execute (e.g., Moisello et al., 2008; Toussaint et al., 2020). Reachability was consistently found to be the critical factor in those studies and we have no reason to expect that the Simon Effect is different in this regard. Nonetheless it cannot be ruled out based on the current results alone that the Simon Effect was modulated by the proximity of stimuli to the hand rather than by the reachability that proximity afforded. An important next step for a future study would be to manipulate reachability in other ways that dissociate reachability from proximity. This could be done, for example, by placing an apparent transparent barrier between the observer and stimuli at otherwise reachable locations (Costantini et al., 2010). If the Simon Effect is driven by functional reachability, then introducing a barrier should reduce it. On the other hand, it is possible that unlike action-affordance congruence effects in which the affordances provided by the stimuli are directly related to the required response, this method of manipulating reachability might not modulate the Simon Effect. Such a finding would provide insight into the nature of the fundamental interdependence between perception and action within the human information processing system. Further, virtual reality expands the range of manipulations that can be introduced and therefore questions that can be addressed. The study by Joy et al. (2022), for example, manipulated participants’ perceptions of what kinds of actions they could and could not make by giving them different avatars. Tools can be provided that extend or limit participants’ capabilities in ways that even violate standard physics. The current finding of perceived reachability, operationalized as perceived distance from the viewer, moderating the Simon Effect provides a foundation for interesting and important follow-up questions to be addressed.

In summary, we found that reachability – operationalized by the distance between objects and the observer – reliably influenced the magnitude of the Simon Effect. This extends previous findings regarding the impact of reachability on task-relevant stimulus-response interactive effects to a task-irrelevant stimulus-response interactive effect, suggesting a fundamental interdependence of perception and action that goes beyond task-based adaptations of processing. These findings present multiple promising directions for future research including testing other methods of manipulating reachability which could reveal differences between the Simon Effect and action-affordance congruence effects.

## Data Availability

All primary data are publicly available on OSF (10.17605/OSF.IO/63FCE).

## Code availability

Code is available upon request.
